# Hydraulic characteristics and carbon metabolism of *Haloxylon ammodendron* under different water–salt content

**DOI:** 10.1093/aobpla/plac042

**Published:** 2022-09-15

**Authors:** Fang Yang, Guanghui Lv, Yadong Qie

**Affiliations:** School of Ecology and Environment, Xinjiang University, Urumqi 830046, Xinjiang, China; Key Laboratory of Oasis Ecology, Ministry of Education, Urumqi 830046, China; Xinjiang Jinghe Observation and Research Station of Temperate Desert Ecosystem, Ministry of Education, Jinghe 833300, China; School of Ecology and Environment, Xinjiang University, Urumqi 830046, Xinjiang, China; Key Laboratory of Oasis Ecology, Ministry of Education, Urumqi 830046, China; Xinjiang Jinghe Observation and Research Station of Temperate Desert Ecosystem, Ministry of Education, Jinghe 833300, China; Guangxi Key Laboratory of Forest Ecology and Conservation, College of Forestry, Guangxi University, Nanning 530004, China

**Keywords:** Carbon metabolism, *Haloxylon ammodendron*, hydraulic characteristics, water–salt content

## Abstract

Drought and salt stress are important abiotic stressors that adversely affect the growth, resistance and survival of plants. *Haloxylon ammodendron* is a strong halophyte, and its hydraulic characteristics and carbon metabolism response to drought and salt stress under natural conditions have not been widely studied. With *H. ammodendron* as the research object, three sample plots with different water and salt contents (high water and high salt, medium salt in reclaimed water, low water and low salt) were selected to determine their water physiology, photosynthetic physiology, carbon physiology and growth status under different water and salt conditions. Studies have shown that drought and salinity affect the hydraulic properties of *H. ammodendron*, reducing the water content and water potential of assimilation branches and secondary branches and increasing the hydraulic conductivity per unit cross-sectional area of biennial shoots. Affected by drought, the content of non-structural carbohydrates (NSCs) in assimilation branches and secondary branches was significantly reduced, and the NSC content of assimilating branches was significantly higher than that in secondary branches. The transportation of NSCs to the secondary branches caused obstacles, and more accumulated in the assimilating branches. In addition, drought reduced *H. ammodendron* photosynthesis and carbon assimilation and limited carbon uptake, resulting in slower growth. Under the influence of drought and salinity, the anisohydric properties of *H. ammodendron* weakened its stomatal regulation ability and made it susceptible to water transport obstacles, but the degree of carbon limitation was relatively small.

## Teaser

Affected by drought, the content of non-structural carbohydrates (NSCs) in assimilation branches and secondary branches was significantly reduced, and the NSC content of assimilating branches was significantly higher than that in secondary branches. The transportation of NSCs to the secondary branches caused obstacles, and more accumulated in the assimilating branches. *Haloxylon ammodendron*photosynthesis and carbon assimilation and carbon uptake were limited, resulting in slower growth. Under the influence of drought and salinity, the anisohydric properties of *H. ammodendron*weakened its stomatal regulation ability and made it susceptible to water transport obstacles, but the degree of carbon limitation was relatively small.

## Introduction

Drought and salinity are the most common coexisting abiotic stress factors that affect plant yield and productivity from the first stage of plant development (Ors *et al.* 2012). Drought and salinization have affected more than 10 % of arable land, and desertification and salinization are spreading rapidly around the world (Fita *et al.* 2015; Daliakopoulos *et al.* 2016; Cuevas *et al.* 2019). The accumulation of dissolved salts in irrigation water leads to progressive ‘secondary’ salinization of irrigated farmland, especially in arid and semi-arid regions, a problem that will worsen in the near future due to the effects of current climate change (Todea *et al.* 2020).

Plants can sense abiotic stress, such as water shortage and salt stress, and appropriately activate their physiological, biochemical and molecular responses to alter metabolism, growth and development (Bartels and Ramanjulu 2005). The study of plant responses and tolerance mechanisms to drought and salt stress is one of the main topics of current botany research.

Drought and salt stress are the main environmental conditions common in arid and semi-arid climates that can reduce soil water potential, lead to osmotic stress in plants (Pivovaroff *et al.* 2021) and cause imbalance of plant ion ratios (Apostol and Zwiazek 2003), affecting membrane systems, stability and enzymatic activity (Boursiac *et al.* 2008; Ruiz-Lozano *et al.* 2012). Drought and salt stress also affect plant hydraulic properties and carbon metabolism (Wan 2010; McDowell 2011; Calvo-Polanco *et al.* 2014). The hydraulic characteristics of plants refer to the maintenance strategy of plant hydraulic functions and the changes in hydraulic parameters caused by this strategy under stress conditions. Carbon metabolism refers to the uptake, transportation, storage and utilization of carbon by plants (McDowell 2011). The safety of plant hydraulic structures and the maintenance of carbon metabolism are necessary conditions to ensure survival. Many studies have shown that hydraulic failure and carbon starvation are the main mechanisms leading to plant death under water scarcity (McDowell 2011; McDowell *et al.* 2013). Different plants have different water regulation strategies, such as the embolism of xylem conduits and the ability to regulate stomata, which will affect the safety of water transport in plants under adversity, thereby affecting their carbon balance. There is an insufficient understanding of the characteristics of water transport and carbon metabolism in response to water and salt stress in plants with different water regulation strategies. Therefore, studying plant water–carbon relationships under water–salt conditions is beneficial for better understanding the ability and strategies of plants to adapt to drought and salt stress.

*Haloxylon ammodendron* is a small super-xerophyte tree, widely distributed in the deserts of Asia and Africa, and is the main constructive species of desert vegetation. It can not only tolerate drought, barrenness and extreme temperatures but also has strong salt tolerance. It is the plant species with the largest individual, the highest biomass and the highest production among the biological components of the desert ecosystem in China. *Haloxylon ammodendron* has the morphological and physiological characteristics of super-xerophytes (Wu *et al.* 2019) and has strong water absorption and water-holding capacity (Xu *et al.* 2007). To adapt to extremely scarce soil water and a strong high-temperature transpiration environment, *H. ammodendron* degenerates into scaly, succulent (juicy), green shoots (assimilative shoots), and the younger green assimilated shoots tend to have a higher metabolic rate.

The Aibi Lake Wetland National Nature Reserve is located in the Bortala Mongolian Autonomous Prefecture in Xinjiang. It belongs to the wetland–desert forest complex ecosystem type, with an average altitude of 189 m. The basin is located in the mid-latitude region of the hinterland of Eurasia, far from the ocean, and the climate is very dry, which is a typical temperate arid continental climate. Annual effective radiation of light is 6.5 × 10^5^ Kcal/m^2^∙a and annual sunshine hours is about 2800 h. The daily average temperature is 6–8 °C, and the frost-free period is 160 days. The annual average precipitation is 90.9 mm, and the annual evaporation is as high as 1600 mm. The annual average relative humidity is 50 %. The annual average number of high wind (wind speed greater than 17 m·s^−1^) days is as high as 165 days, and salt dust and floating and sinking activities are frequent (Gong *et al.* 2016). The vegetation structure of Lake Aibi is a desert forest in an arid area with trees, as the dominant species, associated shrubs and herbs. The special geographical location and climate type enable plants to establish a set of xerophytic and halophytic hydraulic characteristics and carbon metabolism strategies during long-term adaptation to an arid climate and soil salinization.

Considering *H. ammodendron* as the research object in the Lake Aibi basin, by measuring its growth, hydraulic properties, photosynthetic indicators and non-structural carbohydrates (NSCs) under different water–salt gradients, the effects of different water–salt gradients on the hydraulic structure, photosynthetic capacity and changes in carbon metabolism of *H. ammodendron* were analysed. Based on previous studies, we hypothesized that (i) different water–salt gradients affect the water transport and utilization of *H. ammodendron*, but the water regulation strategy of *H. ammodendron* responds according to different water–salt gradients; and (ii) as *H. ammodendron* is in the water regulation strategy of shuttles, different water–salt gradients affect carbon uptake and transport, affecting carbon balance. This study aimed to study the adaptability and water–carbon regulation strategies of *H. ammodendron* in response to different water–salt gradients.

## Materials and Methods

### Study area

**Figure 1.**
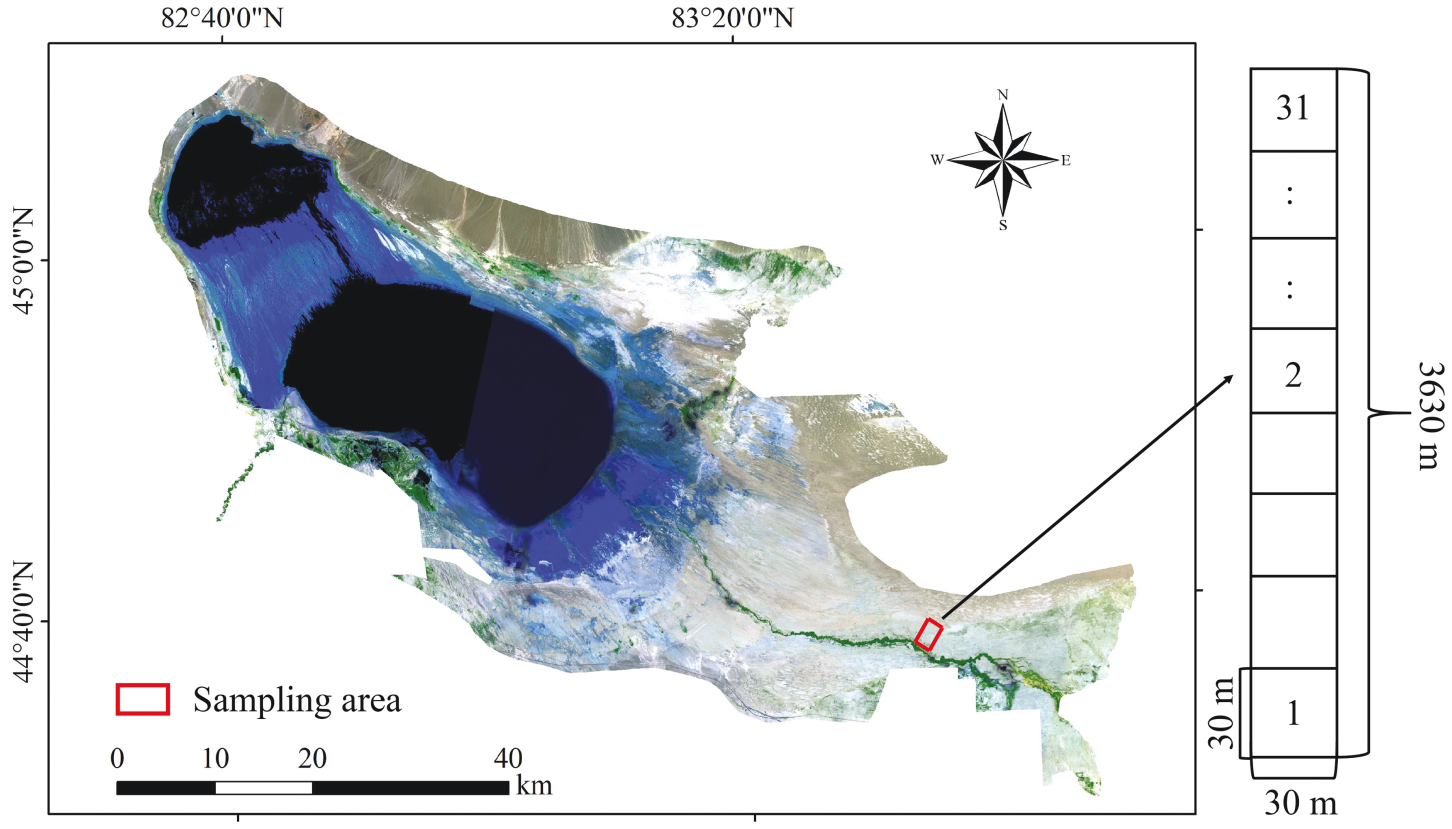
Schematic diagram of the study area and the distribution of sample plots.

The test site was located north of the management station of the East Bridge of the Aibi Lake Wetland National Nature Reserve in Jinghe County, Xinjiang (44°30ʹ–45°09ʹN, 82°36ʹ–83°50ʹE). In 2017, the 30 m × 3630 m standard transect built in 2016 (the southernmost end of the transect is about 500 m away from the bank of the Achikesu River) was investigated and selected for markers of *H. ammodendron* (Fig. 1). In this transect, a 30 m × 30 m standard quadrat was selected every 90 m, for a total of 31 quadrats (Fig. 1). In each quadrat, two mature and healthy *H. ammodendron* individuals with approximately the same diameter at breast height were selected as test plants. If the growth of *H. ammodendron* was not good or not available in the set quadrat, the *H. ammodendron* plants closest to the quadrat were selected horizontally outside the quadrat, totalling 62 plants.

### Sample collection and laboratory measurements

#### Determination of soil moisture, salinity and pH under canopy.

A soil profile with a depth of 50 cm was excavated from the north and south sides of each tested plant with a shovel and divided into the surface layer (0–10 cm), middle layer (10–30 cm) and bottom layer (30–50 cm) for soil sample collection. The soil water content (SWC) of each layer of the soil samples was determined by the drying method, the soil salt content (SSC) of each layer was determined by the conductometric method and the pH of each layer was determined by the potentiometric method (Bao 2000). The arithmetic mean method was used to calculate the mean of the three layers as the measured values of SWC, SSC and pH.

#### Determination of growth indicators.

The plant height and crown width of *H. ammodendron* were measured with a steel tape (accuracy 0.1 cm). Branches from three different directions (south-west, north-west and north-east) were selected outside each canopy, and vernier callipers were used to measure leaf thickness and basal diameter. Since *H. ammodendron* has scaly fleshy leaves with an approximate shape of a cylinder, the leaf area was calculated according to the surface area of the cylinder, and the ratio of leaf dry weight (DW) to leaf area was determined as the specific leaf weight (LMA) (Xiang *et al.* 2013).

#### Determination of water content and water potential of assimilation branches and secondary branches.

In the early morning (3:30–5:00) and at noon (11:30–13:00) (both local times) on consecutive sunny days, the water content (WC) and water potential (*ψ*) were determined. The branches were collected from the middle and upper parts of the canopy on the sunny side, and the *ψ* of each time period was measured (three times for each branch) using the WP4C Dewpoint Potential Meter. At the same time as *ψ* determination, the assimilation branches and their corresponding secondary branches (the definition of branch rank) were classified following Strahler’s system (Mcmahon and Kronauer 1976). The terminal is the zeroth-grade branch of the current year, and the downward branch is, in turn, the first-grade branch. An appropriate amount of sample was separated and cut from different branches and placed a 1/10 000 precision balance (AL204, METTLER TOLEDO, CHN) to determine the fresh weight (FW). Samples were placed in envelope bags and numbered. They were exposed to sunlight for 4 h in the field and brought back to the laboratory after finishing. Finally, they were dried in an oven at 75 °C for 48 h and weighed to determine the DW. Water content was calculated as follows: WC = (FW − DW)/FW.

#### Determination of hydraulic conductivity per unit cross-sectional area of secondary branches.

Branch hydraulic conductivity was measured using a high-pressure flow meter (HPFM, Dynamax Company, Houston, TX, USA). The specific method was as follows: Plant part samples were mixed in water before dawn and equilibrated in the dark for 30 min. When measuring, 1–2 cm was cut from the base of the branch, and part of the epidermis was removed. An appropriate size sealing ring was used with the instrument to connect the sample section to the HPFM, and the instantaneous mode of HPFM was used for measurement (three times for each branch). The HPFM pressure was adjusted to below 7 kPa, and then water was injected into the sample at a pressure increase rate of 3–7 kPa·s^−1^. The water flow rate entering the sample was recorded every 2–4 s. High-pressure flow meter continued to pressurize until about 500 kPa when the water conduction data were completed, and pressurization was stopped. The flow rate and the corresponding pressure were linearly regressed, and the slope of the straight line was determined as the instantaneous hydraulic conductivity (*K*_s_) of the sample.

#### Measurement of the gas exchange index.

In the morning (9:00–11:00) on a sunny day, the gas exchange index was measured. Assimilative branches from three different directions (south-west, north-west and north-east) outside each canopy were selected as three repeated determinations. The LI-6400XT (Li-Cor, Lincoln, NE, USA) photosynthesis system was used to measure the instantaneous photosynthetic rate of the leaves in the red and blue light source leaf chambers. A 2 × 3 cm^2^ red and blue light source (6400-02B) leaf chamber and CO_2_ injection system were used to control the light intensity and CO_2_ concentration. The photosynthetic photon flux density (PPFD) in the leaf chamber was set to 1500, and the reference CO_2_ concentration was set to 400 µmol·mol^−1^. The flow rate was 500 μmol·s^−1^, and the temperature of the leaf chamber was controlled at 30 °C.

During measurement, the leaves were laid flat in the leaf chamber and did not block each other. The sum of the surface areas of the leaves was regarded as the photosynthetically effective area. The measured photosynthetic rate was the maximum photosynthetic rate (*P*_n_). Stomatal conductance (*g*_s_) and transpiration rate (*T*_r_) were recorded, and water-use efficiency (WUE) was determined as the ratio of *P*_n_ and *T*_r_. Since *H. ammodendron* has scaly fleshy leaves with a shape similar to that of a cylinder, the diameter of the leaf was measured with a vernier calliper (0.05 mm), and the total area of the leaf inside the leaf chamber was calculated according to the formula for calculating the surface area of the cylinder. The area should be 1/2 of the calculated area.

#### Determination of NSC content.

Non-structural carbohydrate content is the sum of the soluble sugar (SS) and starch (ST) content. Soluble sugar was determined using the sulfuric acid–anthrone method, and ST content was determined using the perchloric acid method (Mitchel *et al.* 2013). The samples brought back from the field with the calculated WC of assimilated branches and secondary branches were ground into powder, sieved through 80 mesh sieves, numbered and then put into sealed bags for the determination of SS and ST content. The average value of the three repeated determinations was used as the content of SS and ST.

##### Determination of SS content.

A pipette was used to obtain 0.1 mL of SS extract. Five millilitres of anthrone reagent were added to the sample. The samples were shaken gently and boiled in a water bath reaction for 15 min. After the reaction, it was quickly placed in tap water to cool, and then the colorimetric value was measured at a wavelength of 620 nm. This reaction mixture without extract was considered zero as the blank. A standard glucose curve (0, 20, 40, 60, 80, 100 and 120 μg·mL^−1^) was prepared.

##### Determination of ST content:

The precipitate centrifuged with the above SS extract was hydrolysed with perchloric acid and accurately extracted in a boiling water bath for 10 min. It was cooled to room temperature with tap water and centrifuged at 4000 r·min^−1^ for 10 min. The supernatant in the centrifuge tube was taken and transferred to a 50-mL volumetric flask. The absorbance value was measured at a wavelength of 620 nm following the anthrone colorimetry method described previously. Finally, the ST content was calculated according to the standard glucose curve.

### Data analysis

The collected soil samples were measured for SWC, SSC and pH according to the soil layer, and the average SWC, SSC and soil pH were obtained using the arithmetic mean method. The subcanopy soil moisture and salinity were divided into three gradients using *K*-means clustering. One-way analysis of variance (ANOVA) and Tukey’s multiple comparison test (HSD) were used to analyse the change in characteristics of soil environmental factors, hydraulics, photosynthesis, carbon metabolism and growth under the three gradients. The significance level was set to *α* = 0.05. The mean values of early morning water potential, midday water potential and NSC content were compared by an independent sample *t*-test, and the significance level was set to *α* = 0.05. The statistical analysis of the data was done using SPSS v.22.0 (SPSS, Inc., Chicago, IL, USA), and the chart drawing was done in Excel 2016 and Origin 9.0. The relationship between hydraulics, carbon metabolism and shuttle characteristics was analysed by R 4.0.2.

## Results

### Changes in soil environmental factors and water–salt clustering characteristics under the canopy of *H. ammodendron*

**Figure 2.**
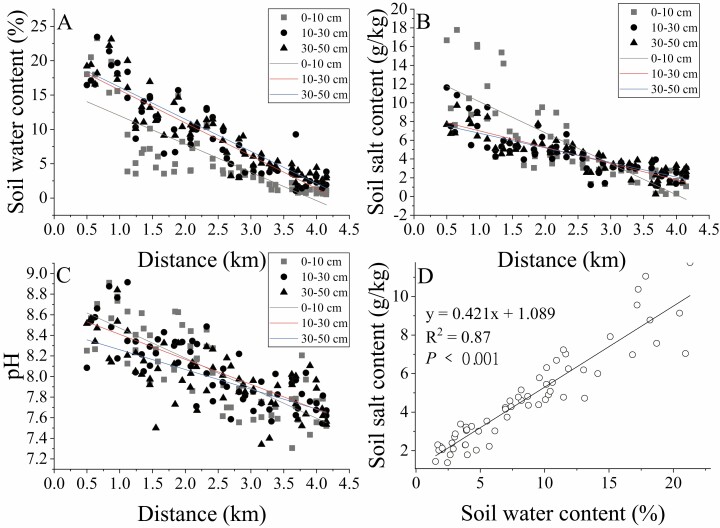
Variation characteristics of soil environmental factors under the canopy of *H. ammodendron*. (A) Variation in soil moisture content. (B) Variation characteristics of soil salinity. (C) Variation in soil pH. (D) Correlation analysis of SWC and salt content.

The three under-canopy soil environmental factors (SWC, SSC, pH) of *H. ammodendron* showed similar variation trends, and the fluctuations became smaller as the distance from the river bank increased (Fig. 2A–C). Within 1.5 km from the river bank, the SWC, SSC and pH values were all high, and these values fluctuated the most. About 3.0 km from the riverbank, the values were obvious and stable. The phenomenon of water and salt migration in the study area was obvious, and farther from the river bank, the lower the WC (18.39–3.46 %) and salt content (9.02–2.48 g·kg^−1^). The correlation analysis between soil water and soil salt content showed that with the increase in soil moisture and soil salinity was significantly linear (*R*^2^ = 0.87, *P* < 0.001, Fig. 2D).

**Figure 3.**
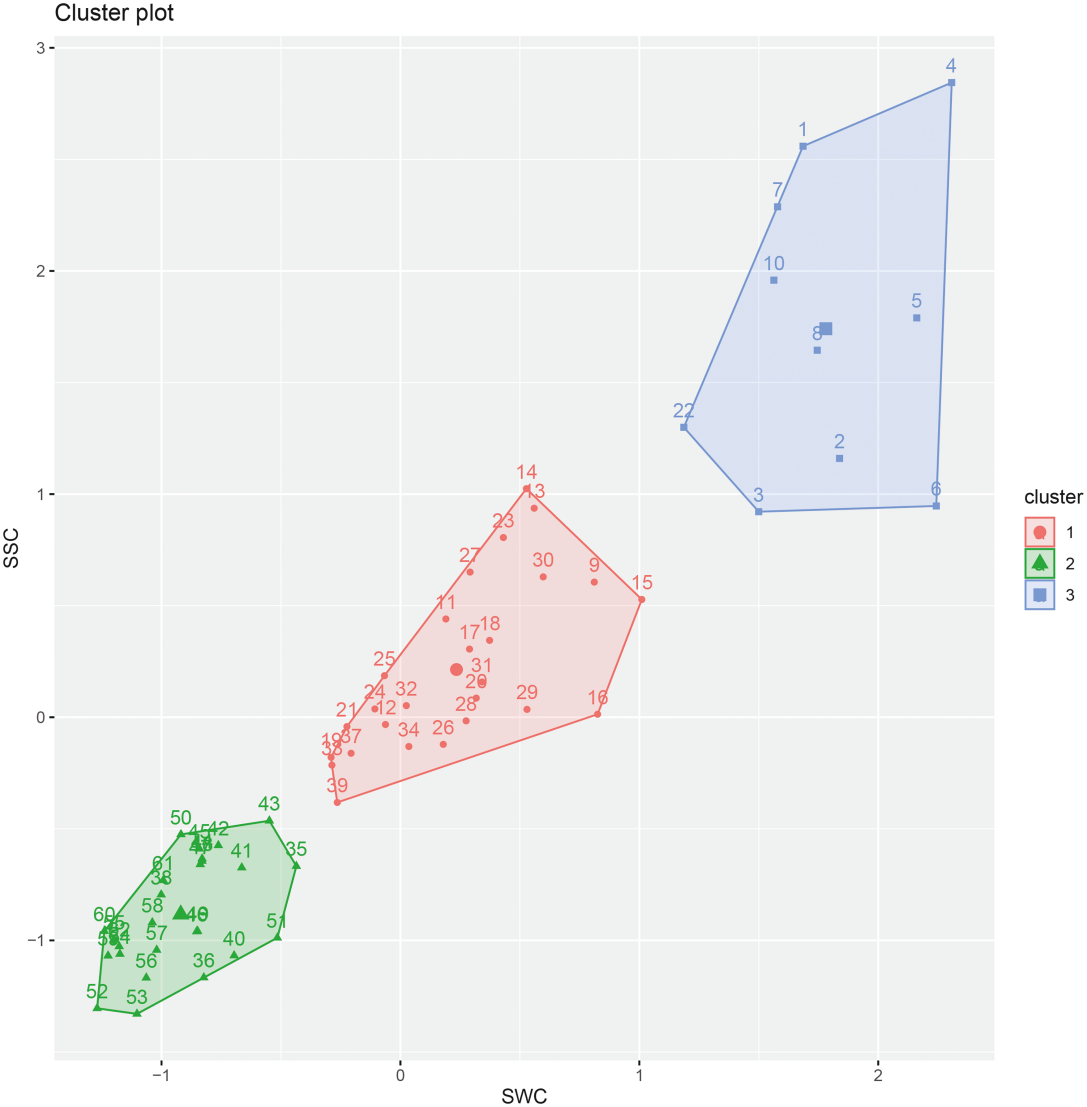
*K*-means clustering results of soil water and salt under the canopy of *H. ammodendron*.

The measured values of soil moisture and salinity under the canopy were clustered and divided into three water–salt conditions according to the *K*-means clustering results (Fig. 3). According to the climatic conditions and soil environment of the Lake Aibi basin, the community structure and vegetation function of the studied transect and salinization and drought attributes of the *H*. *ammodendron* habitat, the transect was divided into three types of soil moisture and salt environments (i.e. high water and high salt, medium water and medium salt and low water and low salt). The first type was mainly distributed in high-water and high-salt (HD) soil environmental characteristics about 1.5 km from the river bank (the first *H. ammodendron* is about 500 m away from the river bank). The second category was the soil environmental characteristics of medium water and medium salt (MD) in the middle zone of the transect (the distance from the river bank was about 1.5–3 km). The third type was distributed at the end of the transect (about 3–4.1 km away from the river bank) and was named low-water and low-salt (LD) soil environmental characteristics.

According to the *K*-means clustering results, the variance analysis of SWC, SSC and soil pH value under different water and salt contents was conducted, and there were significant differences under the three water and salt conditions (*P* < 0.05, Table 1). This indicates that the soil physicochemical environment under the canopy of the tested *H. ammodendron* plants in the observation transect was affected by the interaction between water and salt content.

**Table 1.** Characteristic values of SWC, SSC and soil pH under different water and salt content.

Clustering results	Sample number	SWC (%)	SSC (g·kg^−1^)	Soil pH
High water and high salt	1–8, 10, 22	18.39 ± 1.99a	9.02 ± 1.68a	8.60 ± 0.21a
Medium water and medium salt	9, 11–21, 23–34, 37, 39	9.84 ± 2.03b	5.22 ± 0.95b	8.18 ± 0.28b
Low water and low salt	35, 36, 38, 40–62	3.46 ± 1.28c	2.48 ± 0.61c	7.71 ± 0.18c

The data in the table are the mean ± SD, and different lowercase letters after the values in the same column indicate significant differences in the same index between different stress levels (*P* < 0.05).

### Variation characteristics of the WC of *H. ammodendron* under different water–salt content

**Figure 4.**
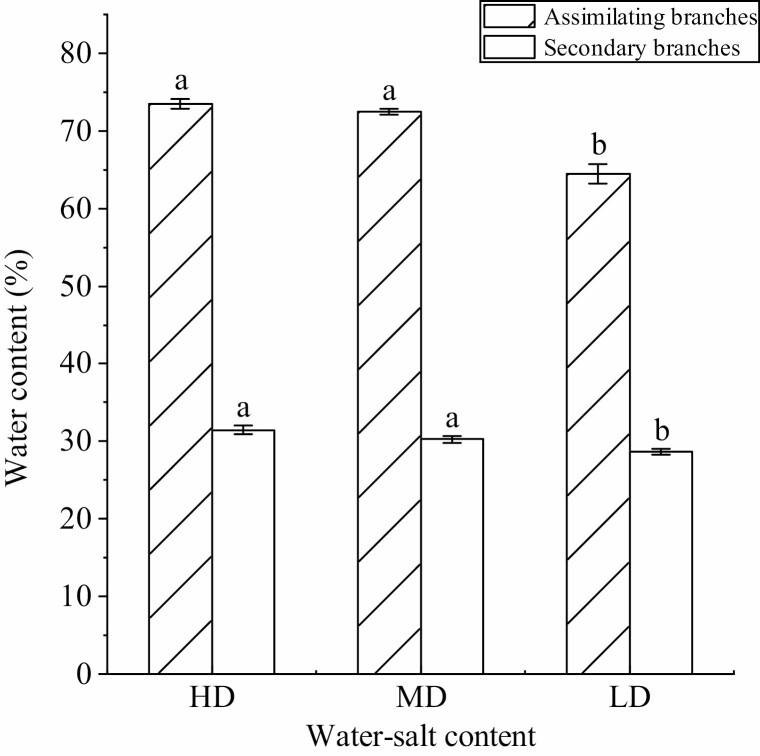
Water content of *H. ammodendron* under different water–salt content (mean ± SD, *N* = 3). Lowercase letters indicate the difference in the WC of assimilation branches and secondary branches between different water–salt content.

The WC of the assimilating branch (WC_A_) and the secondary branch (WC_S_) of *H. ammodendron* (Fig. 4) showed no significant difference between the high-water and high-salt environments and the medium-water and medium-salt environment (*P* > 0.05). The environment with low WC and low salt showed a significant decrease (*P* < 0.05) and reached the lowest value. The WC_A_ was 64.50 ± 1.28 %, with a large decrease range of 12.31 %, WC_S_ was 28.20 ± 1.86 %, and the decrease range was small (8.78 %, *P* < 0.05). WC_A_ was higher than WC_S_ in any water or salt environment.

### Variation characteristics of the water potential of *H. ammodendron* under different water–salt content

**Figure 5.**
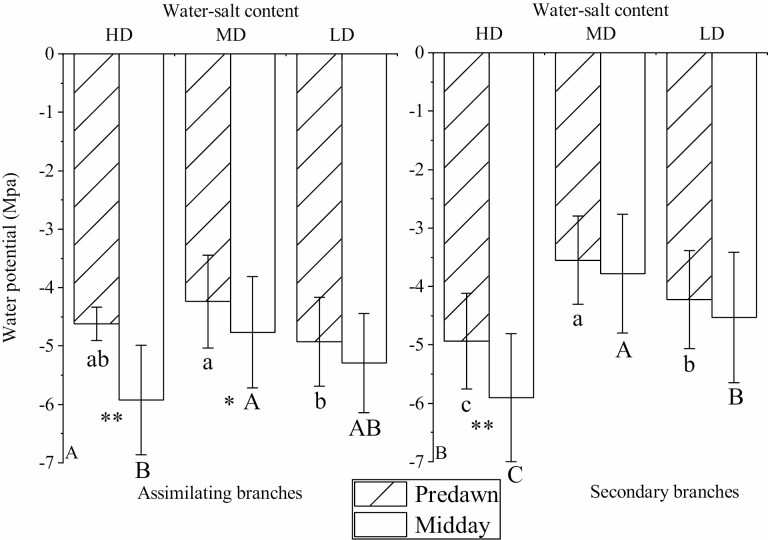
Predawn and noon water potentials of *H. ammodendron* under different water–salt content (mean ± SD, *N* = 3). (A) Predawn and noon water potentials of the assimilation branches. (B) Predawn and noon water potentials of the secondary branches. Lowercase letters indicate the difference in the water potential of assimilation branches and secondary branches between different water–salt content. * and ** represent the significant difference between the predawn and noon water potentials at the levels of 0.05 and 0.01, respectively.

Under the high-water and high-salt environment, the noon water potential (*ψ*_m-a_), the predawn water potential of the secondary branch (*ψ*_p-s_) and the noon water potential (*ψ*_m-s_) of the secondary branch were lower than those in the low-water and low-salt environments (Fig. 5). Compared with the secondary branch, the dawn water potential of the assimilating branch (*ψ*_p-a_) and the water potential difference between them have significant differences in different water and salt environments; that is, the difference between the dawn water potential and noon water potential of the assimilating branches was greater. Under three water and salt environments, the water potential differences of *ψ*_p-a_ and *ψ*_m-a_ were −1.05, −0.69 and −0.50 MPa, respectively, and *ψ*_p-s_ and *ψ*_m-s_ were −0.23, 0.60 and 0.38 MPa, respectively.

### Variation characteristics of hydraulic conductivity per unit cross-sectional area of biennial shoots of *H. ammodendron* in different water–salt content

**Figure 6.**
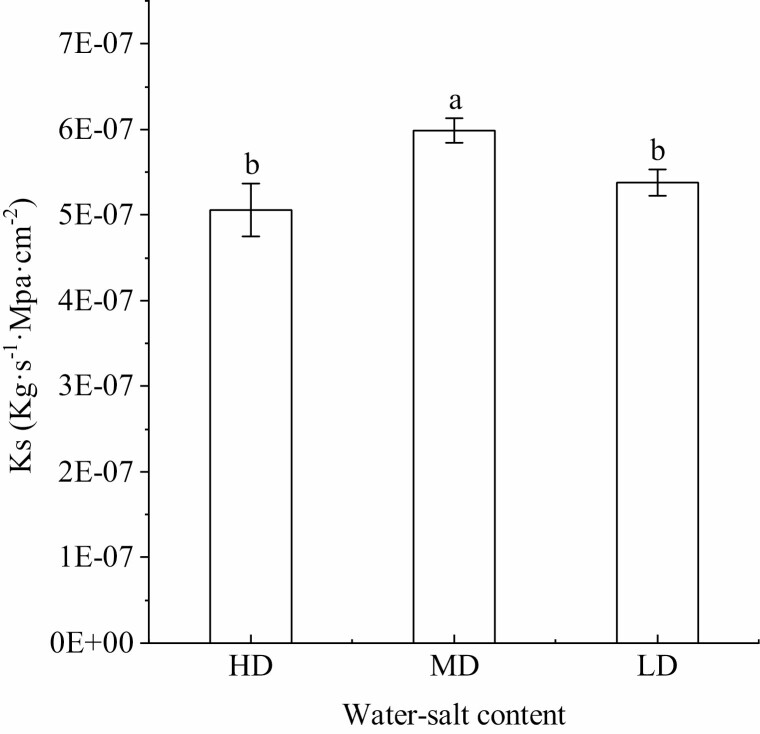
Changes in water conductivity per unit cross-sectional area of the secondary branches of *H. ammodendron* under different water–salt content (mean ± SD, *N* = 3). Lowercase letters represent the difference in the secondary branches’ hydraulic conductivity of *H. ammodendron* among different water–salt content.

The hydraulic conductivity per unit cross-sectional area (*K*_s_) of the secondary branches increased first and then decreased with the decrease of soil water and salt content, and the *K*_s_ in the medium-water–medium-salt environment was significantly higher than that in the high-water–high-salt and low-water–low-salt environments (*P* < 0.05) (Fig. 6). The lowest value of *K*_s_ (5.06E−7 ± 3.10E−8 kg·s^−1^· MPa·cm^−2^) appeared in the high-water and high-salt environment, and the highest value (5.99E−7 ± 1.43E−8 kg·s^−1^·MPa·cm^−2^) appeared in the salt environment with reclaimed water.

### Variation characteristics of photosynthesis of *H. ammodendron* in different water–salt content

**Figure 7.**
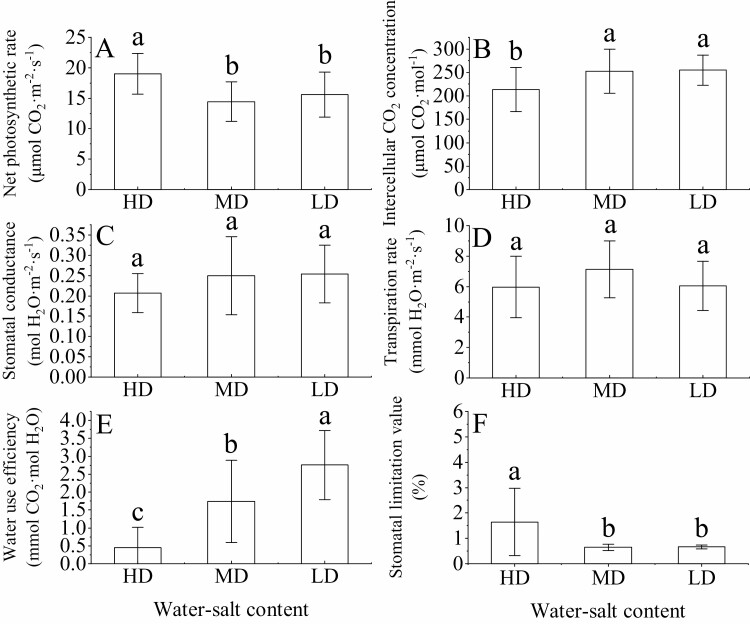
Net photosynthetic rate (A), intercellular CO_2_ concentration (B), stomatal conductance (C), transpiration rate (D), WUE (E) and stomatal limit value (F) of *H. ammodendron* under different water–salt content (mean ± SD, *N* = 3). Lowercase letters represent the differences in the photosynthetic parameters of the assimilation branches among different water–salt content.

Stomatal limit value (*L*_s_) is the main limiting factor for determining plant photosynthesis under water stress. *P*_n_ (19.30 ± 0.85 μmol CO_2_·m^−2^·s^−1^) and *L*_s_ (1.63 ± 1.05 %) in the high-water and high-salt environment were significantly higher than those in the neutral-water or low-water and low-salt content (*P* < 0.05) (Fig. 7A and F). The intercellular CO_2_ concentration (*C*_i_), *g*_s_ and WUE increased with the decrease in the soil water and salt content (Fig. 7B, C and E). *T*_r_ showed a trend of first increasing and then decreasing with the decrease in water and salt content, and *T*_r_ had a minimum value (5.97 ± 0.64 mmol H_2_O·m^−2^·s^−1^) in a high-water and high-salt environment (Fig. 7D).

### Variation characteristics of NSCs of *H. ammodendron* under different water–salt content

**Figure 8.**
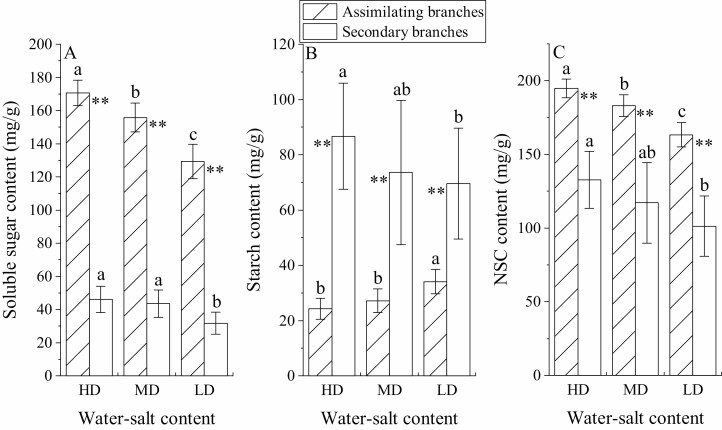
Non-structural carbohydrate content of *H. ammodendron* under different water–salt content (mean ± SD, *N* = 3). A, B and C represent the SS, ST and NSC contents, respectively, of assimilated branches and secondary branches. Different lowercase letters represent the difference in NSC content of assimilated branches and secondary branches in different water–salt content. ** represent the significance of differences in SS, ST and NSC content of assimilated branches and secondary branches at the 0.05 and 0.01 levels, respectively.

The SS and NSC contents in assimilatory branches were the highest in high-water and high-salt environments (170.56 ± 2.42 mg·g^−1^; 194.86 ± 2.01 mg·g^−1^), followed by medium-water–salt environments (155.79 ± 1.69 mg·g^−1^; 182.97 ± 1.45 mg·g^−1^), and the lowest in the low-water and low-salt environment (129.29 ± 2.05 mg·g^−1^; 163.34 ± 1.64 mg·g^−1^) (*P* < 0.05), and the change trend of ST content was the opposite (Fig. 8A–C). The SS, ST and NSC contents in the secondary branches decreased with the decrease in soil water and salt content; that is, the contents were the highest in high-water and -salt environments, which were 46.07 ± 2.51, 86.67 ± 6.07 and 132.74 ± 6.14 mg·g^−1^, respectively (Fig. 8A–C). The SS and NSC contents in assimilation branches were significantly higher than those in secondary branches (*P* < 0.01), while the ST content was significantly lower than that in secondary branches (*P* < 0.01).

### Growth characteristics of *H. ammodendron* under different water–salt content

**Figure 9.**
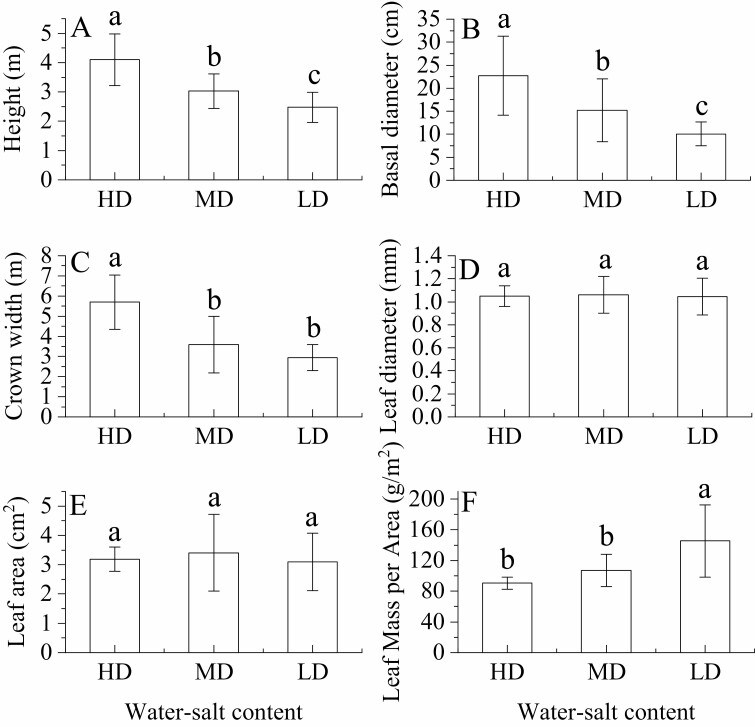
Growth of *H. ammodendron* under different water–salt content (mean ± SD, *N* = 3). A, B, C, D, E and F represent the plant height, base diameter, crown width, leaf diameter, leaf area and specific leaf weight, respectively, of *H. ammodendron*. Lowercase letters represent the difference in *H. ammodendron* growth in different water–salt content.

Under the influence of water and salt stress, the high-water and -salt environments had the highest plant height (4.10 ± 0.28 m) (*P* < 0.05), the largest base diameter (22.73 ± 2.71 cm) (*P* < 0.05) and the largest crown width (33.86 ± 4.96 m^2^), while LMA had a maximum in the low-water and low-salt environments (145.48 ± 9.21 g·m^−2^) (Fig. 9A–C and F). The leaves were the thickest (1.06 ± 0.03 mm) and the leaf area was the largest (3.41 ± 0.26 mm^2^) (*P* > 0.05) in the medium-water–medium-salt environment (Fig. 9D and E).

### Relationship between the assimilating shoot attributes of *H. ammodendron*

With the changes in soil moisture and salinity, there were different correlations among the water physiology, photosynthesis, carbon metabolism and growth of *H. ammodendron* assimilated branches **[see****Supporting Information—Table S1****]**. WC_A_ was significantly positively correlated with SWC (0.626, *P* < 0.001), SSC (0.576, *P* < 0.001), pH (0.531, *P* < 0.001), height (0.332, *P* < 0.001), BD (0.381, *P* < 0.001), CW (0.281, *P* < 0.05), SS (0.710, *P* < 0.001) and NSC (0.696, *P* < 0.001) and significantly negatively correlated with LMA (−0.638, *P* < 0.001), WUE (−0.292, *P* < 0.05) and ST (−0.553, *P* < 0.001). *ψ*_p-a_ was significantly positively correlated with LD (0.259, *P* < 0.05), LA (0.304, *P* < 0.05) and *T*_r_ (0.342, *P* < 0.001). *ψ*_m-a_ was significantly negatively correlated with height (−0.290, *P* < 0.05), BD (−0.292, *P* < 0.05), CW (−0.314, *P* < 0.05) and LA (0.321, *P* < 0.05) and significantly positively correlated with WUE (0.282, *P* < 0.05).

Both SS and NSC had extremely significant positive correlations with SWC, SSC, height, BD and CW and extremely significant negative correlations with LMA and WUE. Starch was significantly negatively correlated with SWC (−0.646, *P* < 0.001), SSC (−0.642, *P* < 0.001), height (−0.313, *P* < 0.05), BD (−0.345, *P* < 0.001), CW (−0.302, *P* < 0.05) and NSC (−0.639, *P* < 0.001) and extremely significantly positively correlated with LMA (0.445, *P* < 0.001) and WUE (0.489, *P* < 0.001).

*P*_n_ was significantly positively correlated with SWC (0.277, *P* < 0.05) and significantly negatively correlated with LMA (−0.251, *P* < 0.05). *C*_i_ was significantly negatively correlated with SWC (−0.280, *P* < 0.05) and CW (−0.284, *P* < 0.05). Water-use efficiency was significantly positively correlated with LMA (0.256, *P* < 0.05) and was significantly negatively correlated with SWC (−0.612, *P* < 0.001), SSC (−0.665, *P* < 0.001), height (−0.559, *P* < 0.001), BD (−0.624, *P* < 0.001) and CW (−0.643, *P* < 0.001).

Height, BD and CW were significantly positively correlated with SWC and SSC, and LMA was significantly negatively correlated with SWC, SSC and LA.

## Discussion

### Effects of different water–salt gradients on the hydraulic characteristics of *H. ammodendron*

Hydraulic characteristics are the water supply and transportation strategies formed by plants to meet the needs of survival under specific environmental conditions (Li and Zhai 2000). Plants adjust and improve their own water transport and water balance by changing their hydraulic properties. The WC of plant leaves can directly reflect the water status of plants and generally indicate the degree of water deficit in plants under drought conditions (Ibarra *et al.* 1988). Excessive soil salt can reduce soil water potential and cause osmotic stress in plants (Munns 2002). In this study, the WC_A_ and WC_S_ in high-water and high-salt environment were significantly higher than those in the low-water and low-salt environment, which indicated that the effect of salt stress was weakened in the presence of high-soil moisture and high-salt content. With the decrease of soil water and salt content, the osmotic stress in the WC of branches also decreased; that is, it was mainly affected by drought stress, resulting in a water deficit.

The decrease in soil water potential causes the negative pressure in the xylem conduit to increase. Under huge negative pressure, plants are prone to cavitation embolism, which destroys the continuity of the water column in the xylem water transport system, restricts the long-distance transport of water within the plant and leads to water mechanical imbalance (Tyree and Zimmermann 2002). In this study, *ψ*_m-a_, *ψ*_p-s_, *ψ*_m-s_ and *K*_s_ were lowest in the high-water and high-salt environment, indicating that a large amount of soluble salts in the soil increased soil osmotic pressure, caused physiological drought in plants and caused their water potential and conductivity. The water rate was reduced, limiting water transport and resulting in poorer water status. The difference between the predawn water potential and the noon water potential reflects the degree of drought tolerance among plant species and individuals within a species. Plants with large differences are anisohydric plants, and plants with basically equal water potentials are isohydric plants (Franks *et al.* 2007). Plants with isohydric potential have a strong stomatal regulation ability, which can reduce water loss through stomatal regulation and make the plant water potential relatively stable. The stomatal regulation ability of non-isohydric plants is weak, so water potential is greatly reduced under water scarcity. Stomatal regulation strategies can affect water transport in plants. Isohydric plants have a strong stomatal regulation ability, which is conducive to maintaining the hydraulic structure, while anisohydric plants have a weak stomatal regulation ability and are prone to hydraulic failure (McDowell *et al.* 2008; Brodribb and Mcadam 2015). Anisohydric plants reduce water potential during midday to maintain gas exchange at the cost of water loss and possible cavitation. Isohydric plants maintain a smaller difference between predawn water potential and noon water potential to conserve water but at the cost of photosynthesis. There are always differences between predawn water potential and noon water potential, but the magnitude of the difference is less for isohydric species or genotypes. Interestingly, *K*_s_ in the high-water–high-salt environment was lowest compared to the reclaimed-water–salt and low-water–low-salt environments, indicating that saline conditions, not water limitations, contribute to differences in branches’ water potential. These differences were still mostly present predawn in the secondary branches, supporting this observation.

### Effects of different water–salt environments on the carbon metabolism of *H. ammodendron*

The stomatal behaviour of plants further affects plant carbon uptake, which in turn affects the carbon balance (McDowell *et al.* 2008; Brodribb and Mcadam 2015). Stomatal closure will limit plant photosynthesis, resulting in reduced carbon uptake, while the carbon consumption of plants to maintain normal physiological activities will not change significantly. Resisting adversity also consumes additional carbon (Adams *et al.* 2013; O’Brien *et al.* 2014). Non-structural carbohydrate is a buffer between plant carbon supply and utilization, which can be used for growth, metabolism and physiological functions (Adams *et al.* 2013), and carbon uptake limitation affects these processes. Furthermore, the carbon uptake limitation affects its hydraulic function, resulting in less carbon availability for growth and limiting rooting, growth and water uptake (Adams *et al.* 2013). The water transport function of plants is also regulated by carbon, and NSCs play an important role in osmotic regulation and embolic repair (Dai *et al.* 2015). Therefore, carbon limitations will further affect the hydraulic structure and stress resistance of plants (Anderegg and Callaway 2012). In this study, with the decrease in soil water and salt content, the photosynthetic rate, growth and NSC content significantly decreased, indicating that *H. ammodendron* was affected by carbon limitation, and carbon limitation further affected the maintenance of its hydraulic structure.

### Interrelationships among *H. ammodendron* hydraulics, carbon metabolism and traits

In arid and semi-arid regions, plants are simultaneously affected by soil salt stress and water limitation, which both affect plant water and carbon metabolism, thereby affecting plant survival (Wan 2010; McDowell 2011). To minimize negative effects, plants adapt to drought and salt stress by reducing physiological and morphological functions (Liu *et al.* 2011; Ge *et al.* 2012). Photosynthesis is one of the main complex processes affected by salinity and drought stress (Chaves *et al.* 2009). In this study, with the decrease in osmotic stress and the intensification of drought stress, *P*_n_ first decreased and then increased, while *g*_s_ increased gradually, which contrasted with the results of Alem *et al.* (2020). This may be because *H. ammodendron* tends to be anisohydric, and its stomatal regulation ability is weak. When affected by drought stress, to ensure the carbon assimilation of photosynthesis, the stomata remain open, resulting in the increase of *g*_s_ and the increase of *P*_n_. Teare *et al.* (1982) believed that plant leaf WUE could be used as an indicator to judge plant WUE, which is usually expressed by *P*_n_/*T*_r_. A high WUE is considered a contributing trait for successful, well-growing and productive plants in early dry and semi-arid environments. In this study, WUE gradually increased with the decrease in soil water and salt content, which indicated that under conditions of severe water deficit, the high WUE of *H. ammodendron* could play a certain role in maintaining the hydraulic structure. Correlation analysis also confirmed this conclusion, as WUE was significantly negatively correlated with SWC, SSC and WC_A_ and significantly positively correlated with *ψ*_m-a_.

Changes in growth under drought and salt stress are an important mechanism by which plants adapt to water and salt stress, and the first measurable physiological effect is drought stress (Seiler and Casell 1990). In general, plants with high LMA have high input cost per unit leaf area, a slow growth rate and strong resistance to (non-)biotic stress. Plants with low LMA have low input cost per unit leaf area, high light utilization efficiency of leaves and fast growth, but their ability to tolerate stress is relatively poor (Poorter and Bongers 2006; Poorter *et al.* 2009). In this study, the plant height, BD and CW of *H. ammodendron* decreased with the decrease of soil water and salt content, while LMA increased. The reduction in plant height contributed to the transfer of water from roots to leaves, with a reduction of 42 % in plant height, 56 % in BD and 73 % in CW, indicating that CW was the most sensitive to drought stress. Plant height, BD and CW were significantly positively correlated with SWC, SSC, WC_A_ and NSC, and significantly negatively correlated with *ψ*_m-a_ and WUE, indicating that water and salt stress affected carbon limitation in *H. ammodendron*, resulting in a slow growth rate. This further affects the maintenance of the hydraulic structure, thereby reducing the WC and water potential.

## Conclusions

Drought and salinity lead to poor water status, limited photosynthesis and reduced growth in *H. ammodendron*. *Haloxylon ammodendron* has a unique carbon–water regulation strategy in response to water–salt stress, preferring anisohydric plants, a weak stomatal regulation ability, unfavourable maintenance of its hydraulic structure and a relatively small degree of carbon limitation, vulnerability to water transport and the impact of obstacles. *Haloxylon ammodendron* has a strong ability to replenish water at night, which makes up for the lack of stomata regulation to a certain extent. Soil salinization is serious in Lake Aibi Basin. Improving soil water conditions can improve the hydraulic structure and carbon metabolism of plants under salinization to a certain extent. Enhanced water management can help plants survive, grow under water and reduce salt stress in arid and semi-arid regions.

## Supporting Information

The following additional information is available in the online version of this article—

Table S1. The relationship between the functional attributes of the assimilation branches of *H. ammodendron*.

plac042_suppl_Supplementary_Tables

## Data Availability

Data from this study are available and can be accessed at the public data repository Dryad. https://datadryad.org/stash/share/E7mbwSDFjvZLmuSzZMKAEoCmaz7Vmkq2Yu9d0EUSg3E

## Notes

**Form & Function. Chief Editor:** Kate McCulloh
